# Improved Pharmacological and Structural Properties of HIV Fusion Inhibitor AP3 over Enfuvirtide: Highlighting Advantages of Artificial Peptide Strategy

**DOI:** 10.1038/srep13028

**Published:** 2015-08-19

**Authors:** Xiaojie Zhu, Yun Zhu, Sheng Ye, Qian Wang, Wei Xu, Shan Su, Zhiwu Sun, Fei Yu, Qi Liu, Chao Wang, Tianhong Zhang, Zhenqing Zhang, Xiaoyan Zhang, Jianqing Xu, Lanying Du, Keliang Liu, Lu Lu, Rongguang Zhang, Shibo Jiang

**Affiliations:** 1 Key Laboratory of Medical Molecular Virology of Ministries of Education and Health, Shanghai Medical College and Shanghai Public Health Clinical Center, Fudan University, Shanghai, 200032, China; 2 National Laboratory of Biomacromolecules, Institute of Biophysics, Chinese Academy of Sciences, Beijing, 100101, China; 3 Lindsley F. Kimball Research Institute, New York Blood Center, New York, New York 10065, USA; 4 Beijing Institute of Pharmacology & Toxicology, 27 Tai-Ping Road, Beijing, 100850, China; 5 Shanghai Public Health Clinical Center and Institutes of Biomedical Sciences, Fudan University, Shanghai, 201508, China; 6 National Center for Protein Science Shanghai, Institute of Biochemistry and Cell Biology, Shanghai Institutes for Biological Sciences, Chinese Academy of Sciences, Shanghai, 201210, China

## Abstract

Enfuvirtide (T20), is the first HIV fusion inhibitor approved for treatment of HIV/AIDS patients who fail to respond to the current antiretroviral drugs. However, its clinical application is limited because of short half-life, drug resistance and cross-reactivity with the preexisting antibodies in HIV-infected patients. Using an artificial peptide strategy, we designed a peptide with non-native protein sequence, AP3, which exhibited potent antiviral activity against a broad spectrum of HIV-1 strains, including those resistant to T20, and had remarkably longer *in vivo* half-life than T20. While the preexisting antibodies in HIV-infected patients significantly suppressed T20’s antiviral activity, these antibodies neither recognized AP3, nor attenuated its anti-HIV-1 activity. Structurally different from T20, AP3 could fold into single-helix and interact with gp41 NHR. The two residues, Met and Thr, at the N-terminus of AP3 form a hook-like structure to stabilize interaction between AP3 and NHR helices. Therefore, AP3 has potential for further development as a new HIV fusion inhibitor with improved antiviral efficacy, resistance profile and pharmacological properties over enfuvirtide. Meanwhile, this study highlighted the advantages of artificially designed peptides, and confirmed that this strategy could be used in developing artificial peptide-based viral fusion inhibitors against HIV and other enveloped viruses.

The envelope glycoprotein (Env) of the human immunodeficiency virus type 1 (HIV-1) includes the gp120 surface subunit and the gp41 transmembrane subunit. As a class I transmembrane protein, gp41 plays a significant role in the membrane fusion and virus entry of HIV-1^1^,^2^. After the binding of the gp120 to the cellular receptors, gp41 undergoes conformation changes after fusion peptide (FP) inserts into the target cell membrane, exposing N-terminal heptad repeat (NHR) trimer, and forming the six-helix bundle (6-HB) structure. 6-HB contains three parallel NHR region and three antiparallel C-terminal heptad repeat (CHR) region, which brings the viral and cellular membranes into close proximity for fusion^1^,^2^,^3^,^4^,^5^. Peptides derived from the HIV-1 gp41 CHR are potent HIV fusion inhibitors^2^,^6^,^7^ (Fig. 1a), which specifically interact with the viral gp41 NHR to form heterogeneous 6-HB to prevent fusogenic gp41 core formation^1^,^2^,^3^,^4^,^5^,^8^,^9^.

As the first-generation HIV fusion inhibitor, T20 (generic name: enfuvirtide; brand name: Fuzeon) was approved by the U.S. FDA to treat patients who fail to respond to the current antiretroviral drugs, such as reverse transcriptase inhibitors and protease inhibitors. However, its low efficacy and short half-life as well as the rapid emergence of T20-resistant viral strains in T20-treated patients have limited its clinical application^10^,^11^,^12^,^13^. It has been reported that the preexisting antibodies in HIV-1-infected patients could block T20-mediated inhibition of membrane fusion by forming T20-antibody complexes^14^. Some antibodies targeting T20’s binding sites in gp120 and gp41 could also attenuate T20-mediated inhibition of cell-cell fusion^15^.

To address these obstacles, many efforts have been made to optimize T20 and gp41 CHR-derived peptides. Some of these peptides have better inhibitory activities against T20-resistant strains and/or longer half-life than T20. However, they still have the problem to cross-react with the preexisting antibodies in the sera of HIV-infected patients because they contain some native CHR sequences. Based on the universal artificial peptide template of 5HRu, we previously designed the artificial peptides of AP1 (PBD-m4HR) and AP2 (PBDtrp-m4HR), and have made preliminary research on their inhibitory activity against HIV-1 Env-mediated cell-cell fusion^16^. In the present study, we designed a new artificial peptide, AP3 (Fig. 1a), aiming to apply the “M-T hook” structure to stabilize the interaction of the artificial peptide with the hydrophobic pocket on the gp41 NHR trimer^17^,^18^. After comprehensively studying its antiviral activity, biochemical property, crystal structure, functional mechanism, *in vivo* half-life and, for the first time, the effect of preexisting antibodies in the sera of HIV-infected patients, we found that the newly designed artificial peptide, AP3, exhibited improved antiviral activity, drug resistance profile and pharmacological properties over T20. Particularly, the preexisting antibodies in the sera of HIV-infected patients did not suppress, but enhanced the anti-HIV-1 activity of AP3. These results suggest that AP3 has potential for development as a new anti-HIV drug and confirm that this strategy can be used for designing artificial antiviral peptides against other enveloped viruses, such as SARS-CoV^19^, MERS-CoV^20^, and paramyxovirus^21^.

## Results

### AP3 inhibited HIV-1 infection with higher potency than T20

**Figure 1:**
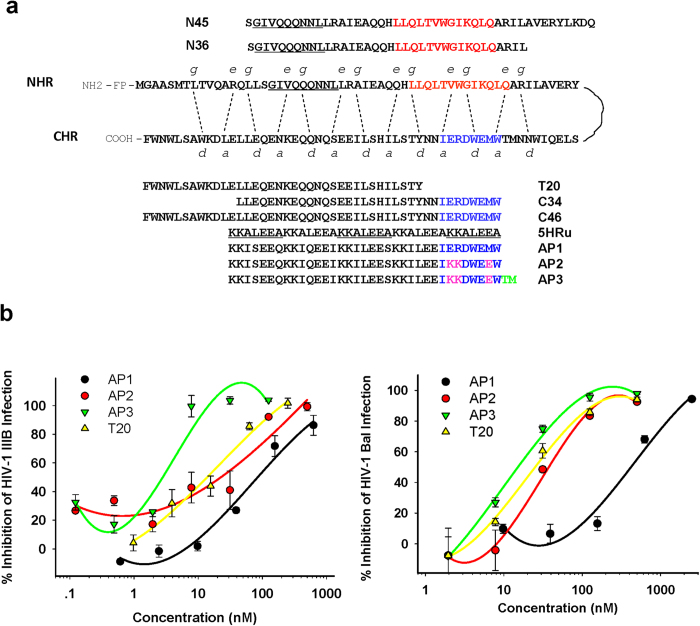
Schematic illustration of the HIV-1 gp41 protein and inhibition of HIV-1 infection by peptides. (**a**) The sequences of gp41 NHR- or CHR-derived peptides. The residues corresponding to the NHR pocket region are marked in red. The residues for the PBD are marked in blue, and the MT-hook residues adjacent to the N terminus of PBD are marked in green. 5HRu peptide consists of 5 copies of artificial sequence template (AEELAKK) underlined. The mutant residues in PBD of AP2 and AP3 were highlighted in pink. (**b**) The inhibitory activity of AP1, AP2, AP3 and T20 on infection by HIV-1_IIIB_ (subtype B, X4) in MT-2 cells (left panel) by HIV-1_Bal_ (subtype B, R5) in M7 cells (right panel). Each sample was tested in triplicate and the experiment was repeated twice. The data are presented as means ± SD.

Our previously designed artificial peptides AP1 and AP2 could inhibit HIV-1 Env-mediated cell-cell membrane fusion^16^. He and colleagues reported that adding two amino acids of Met and Thr to the N-terminus of a CHR-peptide could enhance their anti-HIV-1 activity^17^,^18^. Here we designed a new artificial peptide, AP3, by adding Met and Thr to the N-terminus of AP2 (Fig. 1a). We then compared AP3 with AP1, AP2 and T20 for their anti-HIV-1 activity against divergent HIV-1 strains, including the laboratory-adapted viruses, IIIB (subtype B, X4) and Bal (subtype B, R5), and a series of primary HIV-1 isolates, as well as the T20-resistant strains. As shown in Fig. 1b, AP3 exhibited higher inhibitory activities on infection by HIV-1_IIIB_ and HIV-1_Bal_ strains (IC_50_: 3.06 and 15.09 nM, respectively) than AP1 (IC_50_: 86.25 and 396.14 nM, respectively), AP2 (IC_50_: 23.05 and 49.95 nM, respectively), and T20 (IC_50_: 13.63 and 30.21 nM, respectively). The inhibitory activity of AP3 on infection by divergent primary HIV-1 isolates with distinct genotypes (subtypes A – E and group O) and phenotypes (R5 and X4) was also higher than that of AP2 and T20 (Table 1). While T20 was not effective against T20-resistant HIV-1 strains at the concentration as high as 2,000 nM, AP3 could effectively inhibit infection of these strains with IC_50_ in the range of 13 ~ 90 nM, which was about 2- to 4-fold more effective than AP2 (Table 1). These results indicate that the artificial peptide AP3 has remarkably improved anti-HIV-1 activity against a broad spectrum of HIV-1 strains, including T20-resistant variants, over T20 and the artificial peptides AP1 and AP2.

### The preexisting antibodies in HIV-1-infected patients neither recognized AP3, nor attenuated its anti-HIV-1 activity

**Figure 2:**
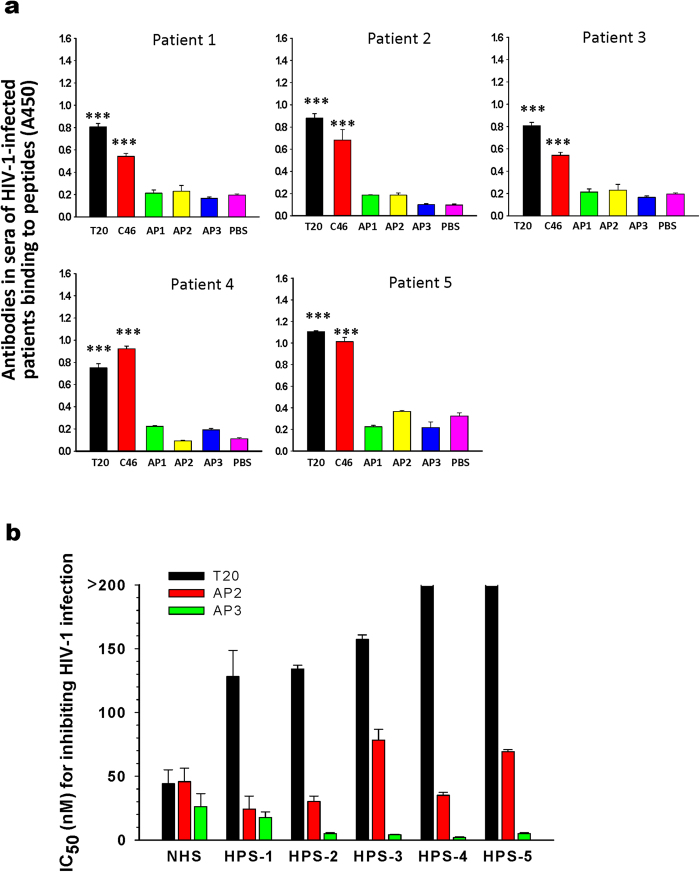
The binding of the preexisting antibodies in the sera of HIV/AIDS patients with the CHR-peptides and the artificial peptides, and the effect of these antibodies on the peptides’ inhibitory activity on HIV-1_IIIB_ infection. (**a**) Peptide-binding activity of the antibodies in the sera of five representative HIV-1-infected patients (1 to 5). (**b**) The IC_50_ values of the AP2, AP3 and T20 for inhibiting HIV-1_IIIB_ infection in the presence of the sera from HIV-1-infected patients (HPS-1 to HPS-5) or normal human serum (NHS). Each sample was tested in triplicate and the experiment was repeated twice. The data are presented as means ± SD. *P < 0.05, **P < 0.01, ***P < 0.001.

Previous studies have shown that the preexisting antibodies in HIV-1-infected patients, including those cross-reacting with T20 and those specific for the binding sites of T20 in gp120 (e.g., the C1 and V3 loop regions) and gp41 (e.g., the NHR domain), could significantly block the fusion inhibitory activity of T20^14^,^15^. Here we investigated the influence of preexisting antibodies against AP3 peptide. As shown in Fig. 2a, both T20 and C46 reacted with the antibodies in sera from five HIV-1-infected patients; however, none of the three artificial peptides AP1, AP2 and AP3 was recognized by the preexisting antibodies. The inhibitory activity of T20 on HIV-1_IIIB_ infection was reduced about 1.9-fold to >3.6-fold in the presence of the sera from HIV-1-infected patients (Fig. 2b and Supplementary Table S1), confirming that the preexisting antibodies in sera of HIV/AIDS patients can attenuate the anti-HIV-1 activity of T20^14^,^15^. However, none of the artificial peptides in the present study showed significant decrease of anti-HIV-1 activity in the presence of patients’ sera. Instead, the antiviral activity of AP3 increased in the presence of antisera from HIV-1-infected patients (Fig. 2b and Supplementary Table S1), suggesting that anti-HIV-1 antibodies actually enhanced the anti-HIV-1 activity of AP3, possibly because the binding of the antibodies to some sites in gp120 or gp41 promote the interaction of AP3 with viral gp41 NHR region.

### AP3 had longer half-life than T20

Although T20 has shown efficacy in inhibiting HIV-1 infection, its major weakness lies in its short half-life in plasma (about 2 h)^22^,^23^,^24^. As a result, T20 has to be administered subcutaneously twice daily at 90 mg per dose, often causing serious injection-site reactions^25^,^26^. Here, we performed pharmacokinetic studies by intravenous administration of AP3, AP2, and T20, respectively, to SD rat at a dose of 1 mg/kg, in order to compare their *in vivo* circulation time. As expected, T20 exhibited a shorter half-life and lower AUC (0–t) from systemic circulation, while AP3 and AP2 demonstrated much higher concentration and longer circulation time (Table 2). The pharmacokinetic profiles of AP3 and AP2 fit a non-compartment model. The pharmacokinetic parameters were calculated with PK Solver. The *in vivo* elimination half-life of AP3 (t_1/2_ = 6.02 h) was about 2.8-fold longer than that of T20 (t_1/2_ = 1.57 h). This result provided the theoretical basis for reducing the injection frequency and dose of the fusion inhibitor, in conjugation with the improved antiviral potency of AP3. Therefore, replacement of T20 with AP3 may significantly reduce injection-site reactions and the drug cost, which would promote the clinical applications of the HIV fusion inhibitor in resource-poor regions or countries.

### AP3 was much more resistant than T20 to proteolytic degradation by proteinase K and rat liver homogenate

**Figure 3:**
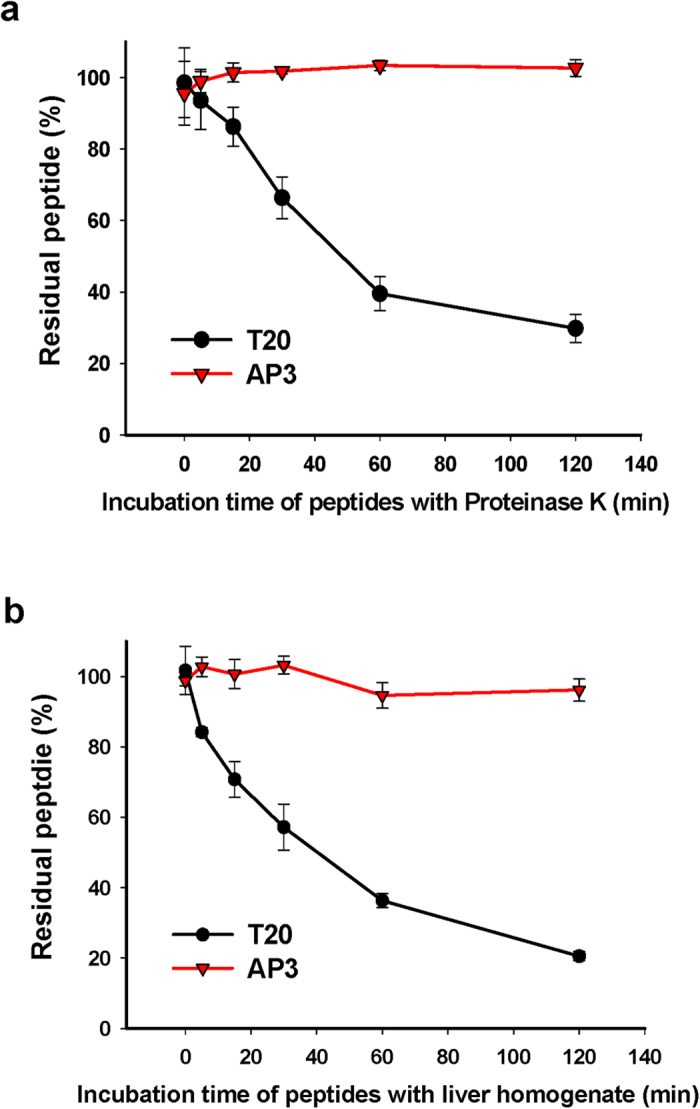
Sensitivity of AP3 and T20 to proteolytic degradation by proteinase K and rat liver homogenate. (**a**) After digestion by proteinase K at pH 7.2 and (**b**) rat liver homogenate, the residual amount of AP3 and T20 was detected by LC-MS analysis. The experiment was performed in triplicate and the data are presented as means ± SD.

We compared the stability of T20 and AP3 in the presence of proteinase K (a broad-spectrum serine proteinase) and rat liver homogenate. After treatment with 20 ng/mL of proteinase K for 2 h at 37 °C, only 29% of the parental T20 peptide remained, as detected by LC-MS analysis. Under the same condition, AP3 retained 100% of its prototype (Fig. 3a). In addition, AP3 showed a significantly enhanced *in vitro* metabolic stability over T20 in the presence of liver homogenate (Fig. 3b). These results indicate that the artificial peptide AP3 is much more resistant to proteolytic degradation than the natural peptide T20, which may contribute to its significant longer *in vivo* half-life than T20 as described above.

### AP3 formed stable α-helical complex and block gp41 6-HB formation

**Figure 4:**
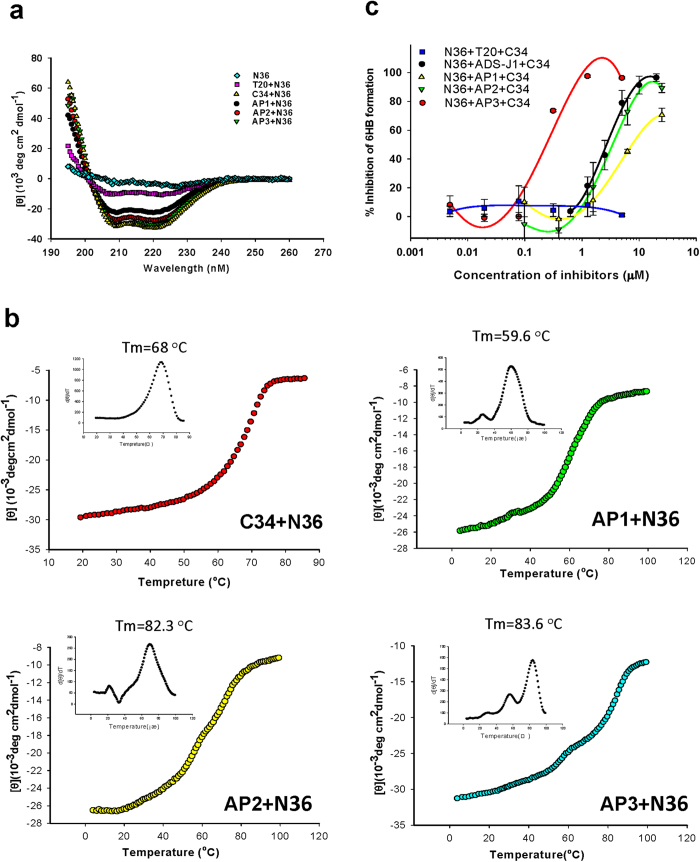
Biophysical properties and 6-HB formation inhibitory activity of AP1, AP2, and AP3. (**a**) α-helicity of the T20/N36, C34/N36, AP1/N36, AP2/N36, and AP3/N36 complexes. All peptides were tested at the final concentration of 10 μM. (**b**) Thermostability (T_m_ value) of the C34/N36, AP1/N36, AP2/N36, and AP3/N36 complexes. (**c**) The inhibition of AP1, AP2, AP3, T20 and ADS-J1 against 6-HB formation between N36 and C34 was detected by ELISA using the 6-HB-specific mAb NC-1. Each sample was tested in triplicate, and the data are presented as means ± SD.

To investigate the antiviral mechanism of AP3, the thermal stability of AP3/N36 complex was compared with that of AP1/N36, AP2/N36, T20/N36, and C34/N36 complexes by circular-dichroism (CD) spectroscopy^27^. Because T20 lacks the pocket-binding domain (PBD), the T20/N36 complex did not show a typical α-helical conformation, in consistence with our previous studies^8^,^9^. Similar to the α-helicity of C34/N36 complex^3^, the AP1/N36, AP2/N36 and AP3/N36 complexes all formed a saddle-shaped negative peak at 208 nm and 222 nm, indicating their α-helical structures (Fig. 4a), suggesting that like the C34 peptide with the native CHR sequence, all three artificial peptides can bind to NHR to form α-helical complex. The α-helix content of AP3/N36, AP2/N36, AP1/N36 and T20/N36 complexes displayed as 77.84%, 69.51%, 65.36% and 36.69%, respectively. The CD thermal denaturation experiment showed a T_m_ value of 68.0, 59.6, 82.3 and 83.6^o^C for C34/N36, AP1/N36, AP2/N36, and AP3/N36 complexes, respectively (Fig. 4b), indicating that the α-helical complex formed by AP3 and N36 is the most stable among the four complexes.

Then we compared the inhibitory activity of AP3 with that of AP1 and AP2 on 6-HB formation between C34 and N36. Since T20 cannot block 6-HB formation^8^,^9^, we used a small-molecule HIV-1 fusion inhibitor, ADS-J1^28^,^29^, to replace T20 as a control of 6-HB inhibition. As expected, ADS-J1 could effectively inhibit 6-HB formation with IC_50_ of 2.75 μM^8^,^9^,^27^,^28^,^29^ . AP3 was highly effective against 6-HB formation in a dose-dependent manner with an IC_50_ value of 0.24 μM, about 30- and 15-fold more potent than AP1 and AP2, respectively (Fig. 4c), confirming that AP3 can potently block gp41 6-HB fusion core formation, thus inhibiting HIV-1 fusion with the target cell membrane.

### Structural basis for the potent fusion inhibitory activity of the artificial peptide AP3

**Figure 5:**
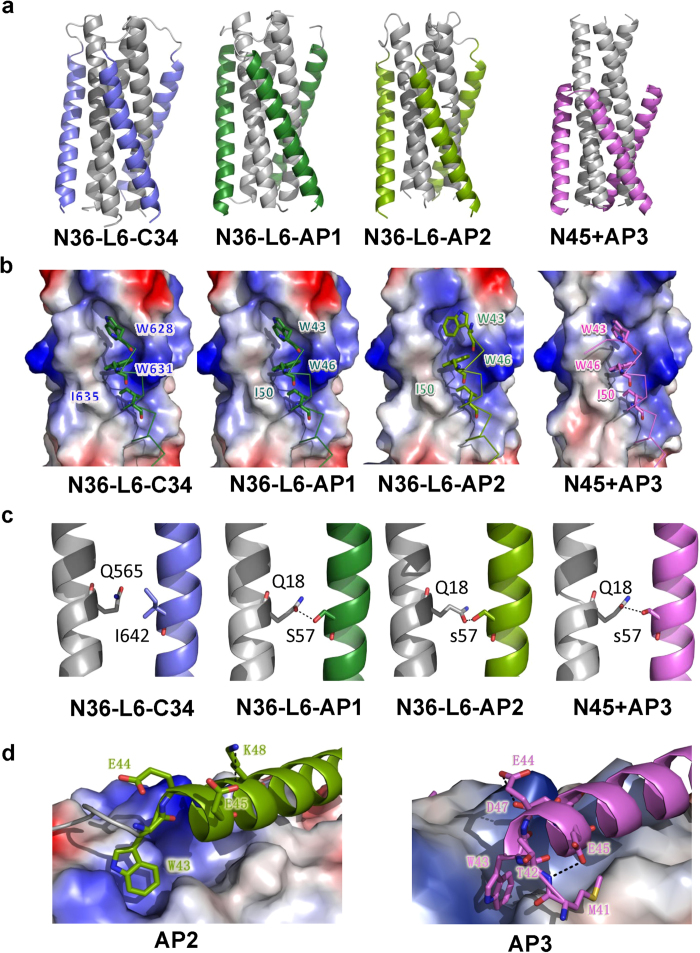
Crystal structure of N36-L6-C34, N36-L6-AP1, N36-L6-AP2, and N45 + AP3 complexes. Colored identically, NHR is in grey, C34 in slate, AP1 in forest, AP2 in split pea and AP3 in violet. (**a**) Cartoon representation of 6-HB structure. (**b**) AP1, AP2 and AP3 are buried inside the hydrophobic pocket formed by NHR. (**c**) AP1, AP2 and AP3 bind to NHR more closely than CHR-peptide C34. The interaction residues are shown in stick representation and are properly labeled. Hydrogen bonds are shown in dark grey dashed lines. (**d**) Different structural characteristics of AP2 and AP3.Colored identically, AP2 is in split pea, and AP3 is in violet. The interaction residues are shown in stick representation and are properly labeled in separate colors.

To elucidate the molecular determinants of these artificial peptides, we successfully solved all three complex structures of AP1/AP2/AP3 peptides binding with gp41 NHR. For AP1 and AP2, an optimized linker “SGGRGG” was used to assemble the NHR and the artificial peptide into a single recombinant protein (N36-L6-AP1 or N36-L6-AP2). However, a similar strategy failed on the crystallization of AP3; therefore, we decided to cocrystallize the synthetic peptide N45 and AP3 peptide, and eventually the complex crystals were obtained. Interestingly, the crystals of three different inhibitors belong to three distinctive space groups: *P*2_1_ for N36-L6-AP1, *R*32 for N36-L6-AP2, and *P*6_3_ for N45/AP3. As expected, the NHR portions in three structures all form a trimeric core, while the AP1, AP2 or AP3 portion folds into a single-helix conformation and binds to NHR-trimer to form a typical 6-HB, similar to that of the HIV-1 gp41 core structure formed by the native CHR peptide C34 and N36 (Fig. 5a). Also, the conserved hydrophobic residues, such as W43, W46 and I50, in the artificial peptides were deeply buried into the hydrophobic grooves formed between each pair of NHR helices, similar to the corresponding residues of W628, W631 and I635 in the native gp41 CHR (Fig. 5b).

AP peptides exhibited better affinity against gp41 natural CHR. In C34, which contains the natural CHR sequence from W628 to L661, no strong interaction between I642 and Q565 in the viral gp41 NHR-CHR complex was found (Fig. 5c). However, in the corresponding sequence (from W43 to K76) of AP1 and AP2, a hydrogen bond was established between S57 (corresponding to I642 in CHR) and Q18 (corresponding to Q565 in NHR) in N36-L6-AP1, N36-L6-AP2 and N45/AP3. Thus, S57 in AP1/AP2/AP3 plays a role in stabilizing the interactions between the artificial peptide inhibitor and its NHR target, resulting in their stronger binding affinity. Moreover, in NHR-CHR, L567 and L568 on two adjacent NHRs form a hydrophobic groove, in which T639 is buried (Supplementary Fig. S1a). However, in N36-L6-AP1, N36-L6-AP2 and N45/AP3, I54 (corresponding to T639 in CHR) can strongly bind to L20 and L21 through fully hydrophobic side chain interactions. Similarly, the interaction of I64 (corresponding to S659 in CHR) with L10 and L11 (corresponding to L567 and L568 in NHR, respectively) in N36-L6-AP1, N36-L6-AP2 and N45/AP3 has been significantly enhanced (Supplementary Fig. S1b).

Like the gp41 CHR helix, the helices of AP1, AP2 and AP3 also have two different sides, a hydrophobic side facing toward the NHR and a hydrophilic one facing outward. It is expected that the enhancement of the hydrophilicity of the exposed side of the inhibitors can increase their antiviral activity and solubility. To achieve this goal, the amino acid residues with hydrophobicity, or low hydrophilicity, like N637, S640, L641 and S644 in CHR, were changed to the amino acid residues with high hydrophilicity, like E52, K55, K56 and E59 in AP1, AP2 and AP3, respectively. Moreover, the hydrophobic residue M629 in CHR was replaced with a hydrophilic residue E44 in AP2 and AP3 (Supplementary Fig. S2). These hydrophilic residues, such as glutamic acid and lysine, can increase the solubility of whole peptide and, hence, stabilize the complex formed by the inhibitor and its target. It has been proved that the EE-KK double salt bridge can stabilize helix conformation^30^. We have identified this kind of interaction between *i* and *i* + 3 or *i* + 4 positions on the three complex structures. In N36-L6-AP1, R48 interacts with E45 and E52 to form a salt bridge network. In N36-L6-AP2, E45 interacts with K48, and E52 binds to K56, while in N45/AP3, K69 binds to E66 (Supplementary Fig. S2). These strong salt bridges formed by the oppositely charged residues stabilize AP peptide conformation, bringing its inhibitory effect into full play.

As previously reported, addition of the “M-T hook” to the CHR peptides C34 and sifuvertide could dramatically improve the anti-HIV-1 activity^17^,^18^. As expected, the N-terminal Met and Thr of AP3 forms a hook-like structure (Fig. 5d). The hydrophobic methionine side chain of M41 accommodates the groove between AP3 and NHR helices, capping the hydrophobic pocket. This interaction leads to a series of conformational changes. The main chain of AP3 at W43 moves 1.91 Å closer to NHR compared to AP2 (Supplementary Fig. S3). The side chain of W43 in AP3 flips around 90 degrees and is buried deeper than that of AP2. The side chain of E44 turns back to interact to D47, but the E45 side chain turns back from K48 and interacts with T42. Therefore, this M-T hook structure could further stabilize the binding between AP3 and NHR target.

## Discussion

Enfuvirtide, also known as T20, was approved by the U.S. FDA as the first HIV entry inhibitor-based antiviral drug for use with other anti-HIV medicines to treat HIV-1 infected adults and children at ages 6–16 years^23^,^31^,^32^ (http://www.fuzeon.com). Although T20 is an indispensable anti-HIV drug for HIV/AIDS patients who have failed to respond to the current antiretroviral therapeutics, its shortcomings have limited its clinical application. T20 has lower anti-HIV activity and shorter half-life than other CHR peptides containing PBD, such as C34 and C38^8^,^9^,^33^. In addition, T20-resistant HIV-1 variants emerged shortly (e.g., 14 days) after its use in patients^34^. Most of the T20-resistant viruses carried mutations in the GIV motif (residues 36-45: GIVQQQNNLL) in the gp41 NHR domain^10^,^34^,^35^,^36^,^37^,^38^. The lack of PBD contributes to the major weaknesses of T20 described above. Since the conserved hydrophobic pocket in the gp41 NHR-trimer plays a critical role in stabilizing the interaction between the gp41 NHR and CHR and formation of the fusogenic 6-HB core^1^,^39^,^40^, the PBD-containing CHR-peptide, like C34, can bind to viral gp41 trimer more strongly and stably, thus possessing more potent anti-HIV activity than T20, a CHR peptide without PBD^8^,^9^. In the absence of PBD, T20 mainly interacts with the middle region of the NHR domain containing the GIV motif. Therefore, a virus with mutations in this motif is generally resistant to T20^10^,^34^,^35^,^36^,^37^,^38^. Compared with other anti-HIV drugs, another weakness of T20 is its cross-reactivity with the preexisting antibodies in HIV-1-infected patients. Besides gp41, T20 could also bind to some regions in gp120. The preexisting antibodies specific for the T20’s binding sites in gp120 and gp41 may indirectly suppress the anti-HIV activity of T20^14^,^15^.

Addition of PBD to the N-terminus of T20, such as T-1249, could significantly improve the anti-HIV-1 potency, half-life and drug-resistance profile^33^,^41^,^42^,^43^. Addition of M-T hook structure to the N-terminus of a PBD-containing CHR-peptides, such as MT-C34 or MT-SFT, could further increase the anti-HIV-1 activity of the corresponding CHR-peptides^17^,^18^. Deletion of the GIV-motif-binding domain from a CHR-peptide, such as CP621-652 and CP32M, is another effective approach to increase the genetic barrier to drug resistance^44^,^45^. However, none of the above approaches is effective in preventing the cross-reaction of T20 with the preexisting anti-gp41 antibodies in HIV/AIDS patients, since the above-modified peptides mainly contain the native sequences of the HIV-1 gp41 CHR domain.

Our previous studies have shown that AP1 and AP2, artificial peptides with non-native protein sequences, could form coiled-coil structure to interact with gp41 NHR and inhibit HIV-1 Env-mediated cell-cell fusion^16^. In the present study, we designed a new artificial peptide, AP3, by adding M-T hook structure to the N-terminus of AP2 (Fig. 1a), followed by investigating the influence of preexisting anti-gp41 antibodies in HIV-infected patients on AP3, using AP1, AP2 and T20 as controls. We demonstrated that sera of HIV-infected patients could bind to T20 and significantly reduce its potency against HIV-1. However, these same serum samples did not interact with the three artificial peptides and hardly impaired their antiviral activity. Surprisingly, the antibodies in the sera could even enhance AP3’s anti-HIV-1 activity (Fig. 2a,b and Supplementary Table S1). These results confirmed, for the first time, that replacement of the native viral sequence in T20 with an artificial sequence is an effective approach to overcome a key shortcoming of T20 whereby its anti-HIV activity could be attenuated by preexisting anti-gp41 antibodies in HIV/AIDS patients. It is worthwhile to explore why the antibodies in the sera is able to enhance the anti-HIV-1 activity of AP3. Our recent study has demonstrated that T20’s anti-HIV-1 activity is enhanced by a non-neutralizing antibody directed against the NHR domain of the HIV-1 gp41^46^. We thus hypothesize that some of the anti-gp41 antibodies in HIV/AIDS patients may bind to a site in NHR domain adjacent to the AP3’s binding region, resulting in increased interaction between AP3 and NHR-trimer and enhanced antiviral activity of AP3.

We then compared the inhibitory activity of AP3 with M-T hook and T20/AP2 without M-T hook on infection by divergent HIV-1 strains. AP3 was more effective than either AP2 or T20 in inhibiting infection by the laboratory-adapted strains and the primary isolates of HIV-1, including those resistant to T20 (Fig. 1b, Table 1). One may question whether AP3 can also induce drug-resistant viruses in patients if it is used in clinics to treat HIV-infected patients. We believe that AP3 is expected to have much higher genetic barrier to resistance than T20 because AP3 contains PBD, while T20 lacks PBD. Dwyer *et al.*^33^ used T2544, a PBD-containing CHR-peptide, to carry out a passaging experiment, using T20 as a control. They demonstrated that T20 could induce a mutant virus with high resistance (81-fold) to T20 in about 1 month, while T2544 failed to induce a resistant strain in more than 2 months in culture. After extending the passaging experiment for almost 8 months, they identified one strain with a weak resistance (8.3-fold) to T-2544, and the related mutation sites were not in the gp41 pocket region, suggesting that the PBD-containing CHR-peptides, including AP3, may have difficulty to induce drug-resistance.

AP3 also had longer half-life than T20 (Table 2), possibly because the artificial peptide AP3 is less sensitive to the proteolytic enzymes than T20 with native viral protein sequence. Removal of the proteolytic enzymes’ cleavage sites in AP3 peptide is expected to further extend its half-life. These results confirmed that replacement of native protein sequence with artificial sequence and addition of the M-T hook to the PBD-containing peptide is a sound strategy for designing HIV fusion inhibitory peptides with improved antiviral activity and pharmacological properties when compared to T20.

Since the three-dimensional structures of AP peptides had not been investigated before the present study, the optimization of these artificial peptide inhibitors could not be performed rationally. Our structural studies of the artificial peptides AP1/AP2/AP3 in complex with NHR showed that AP peptides, just like the CHR peptide C34, could bind to gp41 NHR to form a canonical 6-HB structure (Fig. 5a). It is well known that a deep hydrophobic pocket exists in each groove on the surface of the viral gp41 NHR trimer. The hydrophobic residues I635, W631 and W628 in the gp41 CHR bind with the hydrophobic residues in the wall of this pocket, resulting in the formation of stable 6-HB by the strong interaction between CHR and NHR. This important feature has been well preserved in the AP1/AP2/AP3 6-HB structures (Fig. 5b), which may account for the potent HIV-1 fusion inhibitory activities of these artificial peptides.

A new hydrogen bond, which was established between S57 and Q18 in AP1/AP2/AP3 complexes, does not exist in the viral gp41 CHR-NHR complex, suggesting that S57 may play an important role in stabilizing the interactions between the peptide and NHR, resulting in binding affinities of AP1/AP2/AP3 that are stronger than those of HIV-1 gp41 CHR to NHR. Furthermore, the EE-KK double salt bridge formed between the *i* and *i* + 4 positions in the AP1/AP2/AP3 structures could stabilize helix conformation and increase the inhibitory effect of these peptides. Compared with AP1, triple-site mutations were introduced in AP2 and AP3, *i.e.* M44E, R48K and E49K. Those substitutions not only increase solubility of the peptide, but also trigger a series of rearrangements of certain intrahelical salt bridges to improve the stability of CHR helix structure and HIV-1 fusion inhibitory activity.

M-T hook was previously demonstrated to be an effective step toward increasing the stable interaction between a CHR-peptide and the HIV-1 gp41 pocket^17^,^18^. Therefore, AP2 was further optimized by incorporating Met and Thr at its N-terminus. CD spectroscopy and thermal denaturation results both indicate that the incorporation of M-T hook contribute to the formation of a more stable 6-HB core structure between AP3 (M-T hook-optimized AP2) and N36. In addition, the EE-KK double salt bridge formed between *i* and *i* + 4 positions in the N36-L6-AP3 structure contributed to increased CHR helix and 6-HB stability, resulting in improved potency of AP3, as has been noted in studies of CHR-peptides with EE-KK double mutations^30^,^33^,^47^,^48^. Also, the HIV-1 fusion activity and half-life of AP2 may have been strengthened and extended, respectively, by the addition of M-T hook in the design of AP3.

In conclusion, AP3, an artificial peptide with both PBD and M-T hook structures, exhibited improved anti-HIV-1 activity and drug-resistance profile, as well as prolonged half-life. Moreover, it did not react with the preexisting antibodies in the sera of HIV/AIDS patients. Consequently, its antiviral activity was not significantly affected by these antibodies. Therefore, AP3 shows promise as a candidate for further development as a new HIV fusion inhibitor for clinical use. This study also provides important structure and activity information for the rational design of novel artificially peptide inhibitors. Besides, our results highlighted the advantages of artificially designed peptides and confirmed that this strategy could be widely used in development of artificial peptide-based virus fusion inhibitors against HIV-1 and other enveloped viruses with class I membrane fusion proteins, such as SARS-CoV^19^, MERS-CoV^20^, and paramyxovirus^49^.

## Methods

### Ethics statement

This study did not involve human experimentation; the only human materials used were serum samples obtained from HIV-1-infected individuals with the approval by the Ethics Committee of the Shanghai Public Health Clinical Center, Fudan University (Protocol No. SPHCC-125-2). The methods were carried out in accordance with the approved guidelines. All of these sera samples came from adults; no minor was involved in this study. Written informed consent for the use of the clinical specimens was obtained from all patients involved in this study.

### Peptide synthesis

A panel of peptides (Fig. 1a), including T20, C34, C46, AP1, AP2, AP3, as well as NHR-derived N-peptides, N36 and N45, were synthesized with a standard solid-phase FMOC method, as described previously^8^,^50^. All peptides were acetylated at the N terminus and amidated at the C terminus. The peptides were found to be about 95% pure by HPLC and were identified by mass spectrometry (Perseptive Biosystems, Framingham, MA, USA). Concentrations of the peptides were determined by UV absorbance and a theoretically calculated molar-extinction coefficient based on tryptophan and tyrosine residues.

### Qualification assay

Chromatographic analyses were performed using an ODS-C8 column (5 μm, 100 mm × 2.0 mm ID) kept at ambient temperature. The mobile phase was composed of acetonitrile-water-formic acid in the ratio of 50:50:0.1 (v/v/v) at a flow rate of 0.3 mL/min. The sample injection volume was 10 μL. Acetonitrile was HPLC grade, and other chemical reagents and solvents were analytical grade. A Thermo TSQ Quantum Discovery MAX triple-quadruple tandem mass spectrometer equipped with ESI source (San Jose, CA) and Surveyor LC pump were used for LC-MS analysis. Data acquisition and data processing were performed by using Xcalibur software and LCQuan 2.0 data analysis program (Thermo Finnigan), respectively. Optimized MS parameters were as below: 4800 V spray voltage, 40.0 psi sheath gas pressure, 1.0 psi auxiliary valve flow, and 300 °C of capillary temperature. When running collision-induced dissociation (CID), the pressure was set to 1.5 mTorr. The selected reaction monitoring (SRM) mode was used for AP3 while the selected ion monitoring (SIM) mode was preformed for T20. The following transitions were recorded: m/z 670.5 for AP3, m/z 1498.6 for T20. The masses of synthetic peptides T20, AP1, AP2 and AP3 were determined by MALDI-TOF-MS (Supplementary Fig. S4 and S5).

### Expression and purification of fusion protein N36-L6-AP1 and N36-L6-AP2

Using overlapping PCR, the DNA fragment encoding AP1 or AP2 peptide was attached to the 3′-end of the cDNA of gp41 NHR (“N36”, 546-581), with a short linker (“L6”, SGGRGG) between them. Then, the whole sequence was subcloned into the pET-28a vector (Novagen, USA) with an artificial SUMO-tag between the N-terminal His-tag and the target protein. The pET-28a-SUMO-N36-L6-AP1- or pET-28a-SUMO-N36-L6-AP2-transformed *E. coli* cells were induced by adding 1 mM IPTG and incubating overnight at 16 °C. Fusion protein was purified by Ni-NTA affinity resin (Qiagen, Valencia, CA, USA), and the His-SUMO-tag was cleaved off by Ulp1 enzyme treatment at 4 °C for 2 h. The purified N36-L6-AP1 or N36-L6-AP2 was applied onto a Superdex-75 gel filtration column (GE Healthcare, Piscataway, NJ, USA). Fractions containing N36-L6-AP1 or N36-L6-AP2 trimer were collected and concentrated to different concentrations by ultrafiltration.

### Crystallization, data collection, and structure determination

The fusion protein N36-L6-AP1 was crystallized at 16 °C using the hanging drop, vapor-diffusion method. The drops were set on a siliconized cover clip by equilibrating a mixture containing 1 μl protein solution (25 mg/ml N36-L6-AP1 trimer in 20 mM Tris-HCl pH 8.0 and 150 mM NaCl) and 1 μl reservoir solution (0.1 M Tris-HCl pH 8.5, 32% (w/v) PEG3350, and 0.2 M MgCl_2_) against a 400 μl reservoir solution. After one week, single crystals formed and were flash frozen by liquid nitrogen for future data collection. Fusion protein N36-L6-AP2 was crystallized in a similar way with a different reservoir solution (0.1 M Tris-HCl pH 8.0, 34% (w/v) PEG3350, and 0.2 M MgCl_2_). To obtain the complex crystal of AP3 and NHR, synthesized AP3 was first mixed with peptide N45 at 1:1 molar ratio and then applied onto a Superdex-75 gel filtration column (GE Healthcare, Piscataway, NJ, USA) to isolate the formed 6-HB. Fractions containing N45/AP3 trimer were collected and concentrated to 30 mg/ml, then crystallized at 16 °C using the hanging drop, vapor-diffusion method.The drops were set on a siliconized cover clip by equilibrating a mixture containing 1 μl protein solution (20 mM Tris-HCl pH 8.0 and 150 mM NaCl) and 1 μl reservoir solution (0.2 M Ammonium Sulfate, 0.1 M Bis-Tris pH 6.5, and 25% w/v PEG 3350) against a 400 μl reservoir solution. After 3 days, single crystals formed and were flash frozen by liquid nitrogen for future data collection.

The datasets of N36-L6-AP1 were collected at 100 K at beamline 19-ID of the Advanced Photon Source (Argonne National Laboratory, USA). The datasets of N36-L6-AP2 were collected on an in-house x-ray source (MicroMax 007 x-ray generator, Rigaku, Japan) at the Institute of Biophysics, ChineseAcademy of Sciences. The datasets of AP3/N45 complex crystals were collected at beamline BL-19U1 of the Shanghai Synchrotron Radiation Facility, China. X-ray diffraction data were integrated and scaled using the HKL2000 program^51^. The phasing problem of all three structures was solved by the molecular replacement method using PHENIX.phaser^52^ with a crystal structure of HIV gp41 NHR-CHR (PDB entry: 1SZT) as a search model. The final models were manually adjusted in COOT^53^ and refined with PHENIX.refine^54^. All coordinates were deposited in the Protein Data Bank (N36-L6-AP1: 5CMU; N36-L6-AP2: 5CN0; and N45/AP3: 5CMZ). The statistics of data collection and structure refinement are given in Supplementary Table S2.

### Determination of the cross-reactivity of the native and artificial peptides with the preexisting antibodies in HIV-1-infected patients by sandwich ELISA

A sandwich ELISA was conducted to determine the cross-reactivity of the peptides with the preexisting antibodies in HIV-1-infected patients. T20, C46, AP1, AP2 and AP3 were coated onto the wells of 96-well polystyrene plates (Costar, Corning Inc., Corning, NY) at 10 μg/ml. The wells were then blocked with 1% gelatin, followed by addition of 50 μl of serially diluted sera from HIV-1-infected patients and incubation at 37 °C for 1 h. Then, HRP-labeled goat-anti-human IgG (Abcam, UK) and TMB were added sequentially. A450 was determined with an ELISA reader (Ultra 384, Tecan).

### **Inhibition of HIV-1**_**IIIB**_ **infection by peptides in the presence of sera from HIV-1-infected patients**

Inhibition of peptides on HIV-1_IIIB_ (subtype B, X4)infection in the presence of HIV-1-infected patients’ sera was determined as previously described^55^. Briefly, each peptide was mixed with serially diluted serum from an HIV-1-infected patient at room temperature for 30 min. Next, the mixture of peptide/serum and HIV-1 (100 TCID_50_) were added to MT-2 cells (1 × 10^5^/ml) in RPMI 1640 medium containing 10% FBS. After incubation at 37 °C overnight, the culture supernatants were replaced with fresh culture medium. On the fourth day post-infection, culture supernatants were collected for detection of p24 antigen by ELISA.

### CD Spectroscopy and Thermal Midpoint Analysis

The secondary structure of AP1, AP2 or AP3 peptides mixed with N36 was analyzed by CD spectroscopy as previously described^56^. Briefly, each peptide or peptide mixture was dissolved in phosphate-buffered saline (PBS: 50 mM sodium phosphate and 150 mM NaCl, pH 7.2) at the final concentration of 10 μM and incubated at 37 °C for 30 min before cooling down to 4 °C. The CD spectra of each sample were acquired on a Jasco spectropolarimeter (Model J-815, Jasco Inc., Japan) at 4 °C using a 5 nm bandwidth, 0.1 nm resolution, 0.1 cm path length, and an average time of 5.0 sec. Spectra were corrected by the subtraction of a blank corresponding to the solvent composition of each sample. Thermal midpoint analysis was used to determine the temperature at which 50% of the 6-HB formed by the CHR and NHR would decompose. It was monitored at 222 nm from 4 °C to 98 °C by applying a thermal gradient of 5 °C/min. The melting curve was smoothed, and the midpoint of the thermal unfolding transition (Tm) values was calculated using Jasco software utilities as described above.

### Inhibition of gp41 six-helix bundle formation by sandwich ELISA

Inhibition of gp41 six-helix bundle formation by a testing peptide was determined with a sandwich ELISA described previously^57^. Briefly, a testing peptide (ADS-J1 as a control) at graded concentrations was preincubated with peptide N36 (1 μM) at 37 °C for 30 min, followed by the addition of peptide C34 (1 μM) and incubation at 37 °C for another 30 min. The mixture was added to a 96-well polystyrene plate (Costar, Corning Inc., Corning, NY) precoated with anti-N36/C34 antibodies (2 μg/ml) purified from mouse antisera specifically against the gp41 six-helix bundle^58^. Then, mAb NC-1, HRP-labeled rabbit-anti-mouse IgG (Sigma), and TMB were added in order. A450 was determined by an ELISA reader (Ultra 384, Tecan).

### Inhibition of peptides on laboratory-adapted HIV-1 infection

Inhibition activities of AP1, AP2, and AP3 on HIV-1 infection were determined as previously described^57^. For inhibition of HIV-1_IIIB_ (subtype B, X4) infection,100 TCID_50_ of the virus was added to 1 × 10^5^/ml MT-2 cells in RPMI 1640 medium containing 10% FBS in the presence or absence of the test peptide overnight. Then, the culture supernatants were changed to fresh media. On the fourth day post-infection, culture supernatants were collected for detection of p24 antigen by ELISA. For inhibition of infection by the HIV-1 strain Bal (subtype B, R5), M7 cells (1 × 10^5^/ml) were precultured overnight and infected with Bal at 100 TCID_50_ in the presence or absence of the test peptide or protein overnight. Then, the culture supernatants were changed to fresh media. On the fourth day post-infection, the culture supernatants were discarded, and fresh media were complemented again. The supernatants were collected on the seventh day post-infection and tested for p24 antigen by ELISA as previously described^55^. The percent inhibition of p24 production was calculated.

### Analysis of the half-life of peptide inhibitors

Four male SD rats weighing approximately 200 g each were obtained from the Shanghai Medical School Animal Center and were used for the half-life assay. Animals were treated in accordance with the Animal Welfare Act and the “Guide for the Care and Use of Laboratory Animals” (NIH Publication 86-23, revised 1985). Either AP2 or AP3 was intravenously injected at the concentration of 1 mg/ml. After injection, blood samples were acquired from rat orbit at several time points (8 and 30 min and 1.5, 3, 6, 9, 12, and 24 h after peptide injection) and placed in clean tubes. To study the pharmacokinetics of AP2 and AP3 in rats and provide experimental evidence for the possible pharmacokinetics in human, a double-antibody sandwich ELISA method was established for rapid determination of AP2 and AP3 in rat plasma. Briefly, 96-well polystyrene plates (Costar, Corning Inc., Corning, NY) were precoated with antibody against AP2 or AP3 (5 μg/ml) purified from rabbit anti-sera^59^. They were then preincubated with serum samples diluted 20 times at 37 °C for 1 h, followed by the addition of anti-AP2 or anti-AP3 antibody (1:1000) purified from mouse antisera specifically against AP2 or AP3^59^ at 37 °C for another 1 h. Then, HRP-labeled rabbit-anti-mouse IgG (Sigma, USA) and TMB were added in order. Absorbance at 450 nm was determined by an ELISA reader (Ultra 384, Tecan). The standard peptide parameters were obtained first. Then, the plasma peptide concentrations were determined as a function of time, and the half-life was calculated by using PK Solver for Microsoft Excel to obtain pharmacokinetic parameters.

### Assessment of sensitivity of peptides to proteolytic digestion by proteinase K and proteolytic enzymes in liver homogenate

The peptides (10 μg/mL) were prepared in PBS pH 7.2 containing 20 ng/ml proteinase K. The resulting mixture were incubated at 37 °C in a water bath and taken out at different time intervals (0, 5, 15, 30, 60, 120 minutes), followed by quenching the samples with ethyl alcohol and quantitating the peptides by LC-MS analysis as described above.

To test the sensitivity of peptides to the proteolytic enzymes in liver homogenate, 3 male SD rats (250 ± 20 g) were sacrificed under anesthesia. The whole liver was quickly removed from each rat, washed in ice-cold PBS (50 mM, pH 7.2), weighed and cut into small pieces, which were resuspended in PBS to 100 mg wet liver tissue/2.5 ml PBS. The samples were pooled and homogenized, followed by centrifugation at 9,000 g for 20 min at 4 °C. The supernatants were collected. The test peptides were added to the liver homogenate at a final concentration of 10 μg/ml. The resulting mixture was incubated 37 °C in a water bath, and the residue peptides in the mixture were quantitated as described above.

## Additional Information

**How to cite this article**: Zhu, X. *et al.* Improved Pharmacological and Structural Properties of HIV Fusion Inhibitor Ap3 over Enfuvirtide: Highlighting Advantages of Artificial Peptide Strategy. *Sci. Rep.* **5**, 13028; doi: 10.1038/srep13028 (2015).

## Supplementary Material

Supplementary Information

## Figures and Tables

**Table 1:** Inhibitory activity of peptides on infection by HIV-1 primary isolates and T20-resistant HIV-1 variants.

	**IC50 (nM)**		
**HIV-1 strains**	**AP2**	**AP3**	**T20**
Primary HIV-1 isolates (subtype)			
92UG029 (A)	39.9 ± 4.2	7.1 ± 1.7	8.6 ± 1.7
94US_33931N (B)	38.0 ± 12.5	10.2 ± 0.7	16.2 ± 2.8
93IN101 (C)	35.6 ± 0.9	16.3 ± 1.5	16.8 ± 6.5
92UG024 (D)	34.5 ± 2.3	16.5 ± 0.2	15.5 ± 3.5
92TH009 (A/E)	50.0 ± 4.5	27.8 ± 5.5	14.2 ± 12.4
NP1525 (A/E)	65.6 ± 10.9	32.3 ± 8.8	29.7 ± 6.9
93BR020 (F)	42.7 ± 1.4	19.9 ± 0.0	24.5 ± 3.6
BCF020 (Group O)	47.1 ± 2.1	21.9 ± 0.1	30.8 ± 0.5
T20-resistant HIV-1 variants			
V38A	41.362 ± 8.652	12.635 ± 2.658	943.04 ± 46.424
V38A/N42D	63.524 ± 6.365	31.254 ± 6.621	>2,000
N42T/N43K	72.654 ± 7.525	35.201 ± 12.524	>2,000
V38E/N42S	87.365 ± 5.256	42.369 ± 11.584	>2,000
V38A/N42T	262.35 ± 5.632	89.982 ± 21.525	>5,000

**Table 2:** Pharmacokinetic parameters of AP2, AP3 and T20 following intravenous administration at 1 mg/kg in male SD rats (n = 2).

	**Peptides**		
**Parameters**	**AP3**	**AP2**	**T20**
AUC (0-t) (μg/ml/h)	1205.57	412.36	502.77
Cmax (μg/ml)	289.57	667.43	314.80
t_1/2_(h)	6.02	5.25	1.57
MRT (0-inf_obs) (h)	8.69	7.57	2.27
