# Supplementary material for: Improved Pharmacological and Structural Properties of HIV Fusion Inhibitor AP3 over Enfuvirtide: Highlighting Advantages of Artificial Peptide Strategy
Source: Sci Rep. 2015 Aug 19;5:13028. doi: 10.1038/srep13028 (PMC4541410; doi:10.1038/srep13028)
Supplement: Supplementary Information [file srep13028-s1.pdf]

Supplementary information

Improved Pharmacological and Structural Properties of HIV Fusion Inhibitor AP3 over Enfuvirtide: Highlighting Advantages of Artificial Peptide Strategy

Running title: Artificial peptide-based HIV fusion inhibitor

Xiaojie Zhu[1,†], Yun Zhu[2,†], Sheng Ye[2,†], Qian Wang[1], Wei Xu[1], Shan Su[1], Zhiwu Sun[1], Fei Yu [1,3], Qi Liu[1], Chao Wang[4], Tianhong Zhang[4], Zhenqing Zhang[4], Xiaoyan Zhang[1,5], Jianqing Xu[1,5], Lanying Du[1,3],Keliang Liu[4], Lu Lu[1,*], Rongguang Zhang[2,6,*] & Shibo Jiang[1,3,*]

[1]Key Laboratory of Medical Molecular Virology of Ministries of Education and Health, Shanghai Medical College and Shanghai Public Health Clinical Center, Fudan University, Shanghai, 200032, China [2]National Laboratory of Biomacromolecules, Institute of Biophysics, Chinese Academy of Sciences, Beijing, 100101, China [3]Lindsley F. Kimball Research Institute, New York Blood Center, New York, New York 10065, USA [4]Beijing Institute of Pharmacology & Toxicology, 27 Tai-Ping Road, Beijing, 100850, China [5]Shanghai Public Health Clinical Center and Institutes of Biomedical Sciences, Fudan University, Shanghai, 201508, China [6]National Center for Protein Science Shanghai, Institute of Biochemistry and Cell Biology, Shanghai Institutes for Biological Sciences, Chinese Academy of Sciences, Shanghai, 201210, China.

†These authors contributed equally to the work.

*To whom correspondence should be addressed. E-mail: lul@fudan.edu.cn (L. Lu); rzhang@sun5.ibp.ac.cn (R.-G. Zhang); shibojiang@fudan.edu.cn (S. -B. Jiang).

Supplementary Tables contains: **Supplementary Table S1:**

Table S1. Inhibitory activity of T20, AP1, AP2 and AP3 on HIV-1 IIIB infection in the presence or absence of sera from HIV-1-infected patients.

	T20		AP1		AP2		AP3	
	IC_{50} (nM)	Fold change	IC_{50} (nM)	Fold change	IC_{50} (nM)	Fold change	IC_{50} (nM)	Fold change
N	44.1±10.9	1.0	111.5±29.0	1.0	45.8±10.5	1.0	26.2±10.2	1.0
HPS-1	128.1±20.5	2.9	87.4±54.8	0.8	24.3±10.2	0.5	17.7±4.3	0.7
HPS-2	134.0±44.9	3.0	70.1±44.9	0.6	30.3±4.2	0.7	5.1±0.7	0.2
HPS-3	157.2±22.0	3.6	164.8 ±26.6	1.5	78.3±8.6	1.7	4.2±0.4	0.2
HPS-4	>200	>4.6	89.5 ±20.6	0.8	35.1±2.3	0.8	2.0±0.6	0.1
HPS-5	>200	>4.6	68.5 ±22.6	0.6	69.3±3.2	1.5	5.1±0.8	0.2

The samples were tested in triplicate, and the data were presented in mean ± SD. T20 was included as a control.

Supplementary Table S2:

Table S2. N36-L6-AP1, N36-L6-AP2 and N45/AP3 diffraction data and refinement statistics

Crystallographic data	N36-L6-AP1	N36-L6-AP2	N45/AP3
d_{min} (Å)	2.10(2.10-2.15)	1.90(1.90-1.93)	2.60(2.60-2.64)
Wavelength (Å)	0.97937	1.5418	1.03317
Measured reflections	44242	64372	61523
Average redundancy	3.6	10.5	7.7
Mean I/ (I)	16.9	28.9	27.2
Completeness (%)	99.1(99.4)	100(100)	99.9(100)
R_{merge} [a]	0.082(0.505)	0.084(0.592)	0.122(0.382)
Refinement statistics			
Bragg spacing (Å)	2.11-31.03	1.90-31.78	2.57-37.98
Space group	$P2_1$	R32	$P6_3$
Cell parameters			
a (Å)	36.62	36.19	44.40
b (Å)	58.81	36.19	44.40
c (Å)	51.44	286.00	227.90
Reflections in working set	10388	7025	7574
Reflections in test set	1140	787	340
R_{cryst} [b]	0.2115	0.1961	0.2448
R_{free} [c]	0.2440	0.2324	0.2642
r.m.s.d. bonds (Å)	0.006	0.019	0.004
r.m.s.d. angles (°)	0.838	1.561	0.471
Average *B*-factor ($Å^2$)	42.24	28.7	67.88
No. of waters	71	68	37

Values in parentheses indicate the corresponding statistics in the highest resolution shell.

[a] R_{merge} = (I_i – ‹I_i›|)/I_i|, where I_i is the integrated intensity of a given reflection.

[b] R_{cryst} = (||F_o| – |F_c||)/|F_o|, where F_o and F_c denote observed and calculated structure factors, respectively.

[c] R_{free} is equivalent to R_{cryst}, but calculated using randomly chosen 10% reflections as the test set, which were excluded from the refinement process.

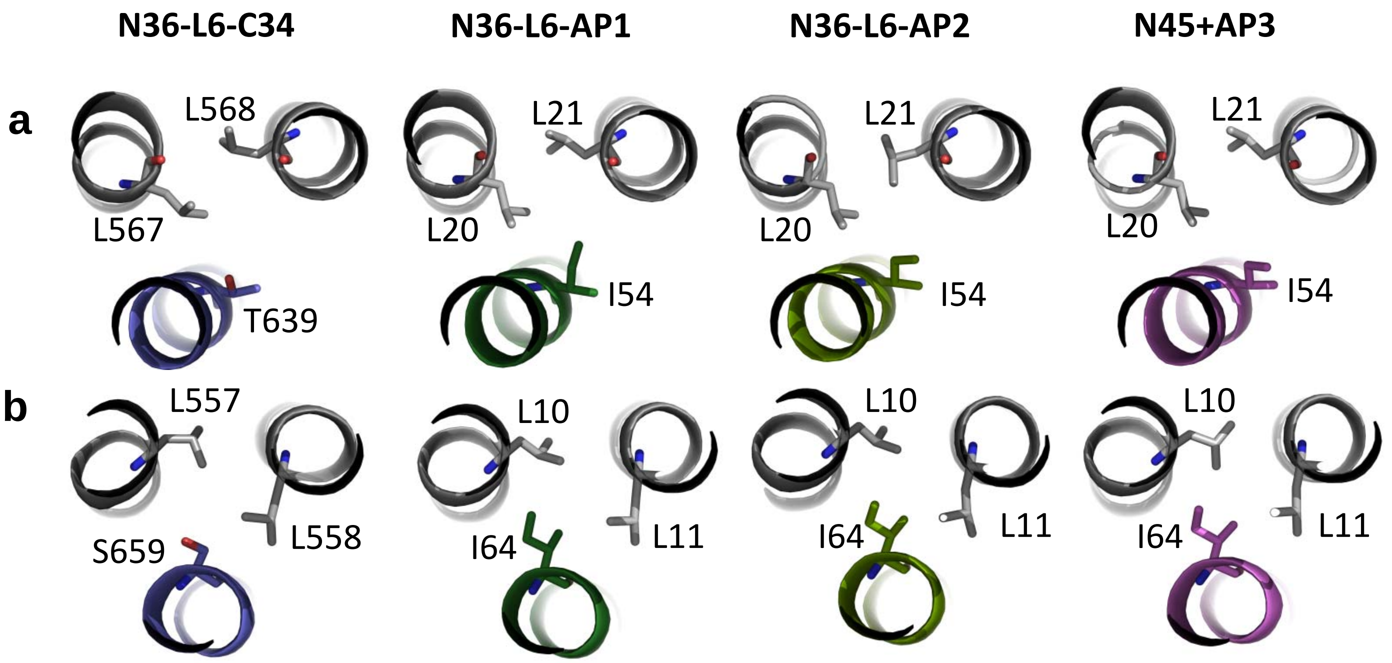

Figure S1 | AP1, AP2 and AP3 bind to NHR more closely than CHR. Cartoon representations of NHR-CHR, N36-L6-AP1, N36-L6-AP2, and N45+AP3 crystal structure are shown in a separate column. Colored identically, NHR is in grey, CHR in slate, AP1 in forest, AP2 in split pea and AP3 in violet. The key residues are shown in stick representation and are properly labeled.

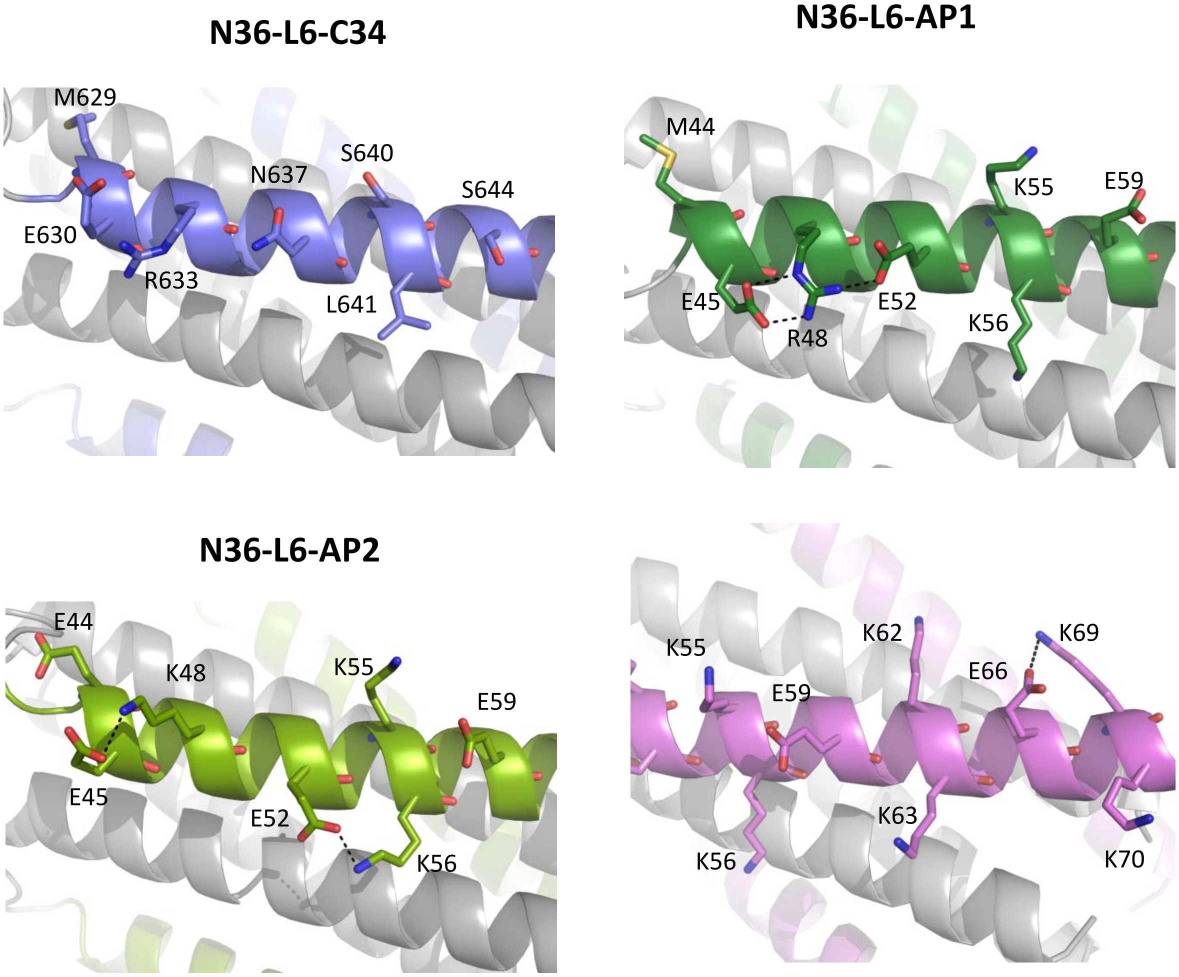

Figure S2 | Surface-exposed residues of AP1, AP2 and AP3. NHR-CHR, N36-L6-AP1, N36-L6-AP2, and N45+AP3 are all shown in cartoon representations. Colored identically, NHR is in grey, CHR in slate, AP1 in forest, AP2 in split pea and AP3 in violet. Key residues are shown in stick representation and are properly labeled. Hydrogen bonds are shown in dark grey dashed lines.

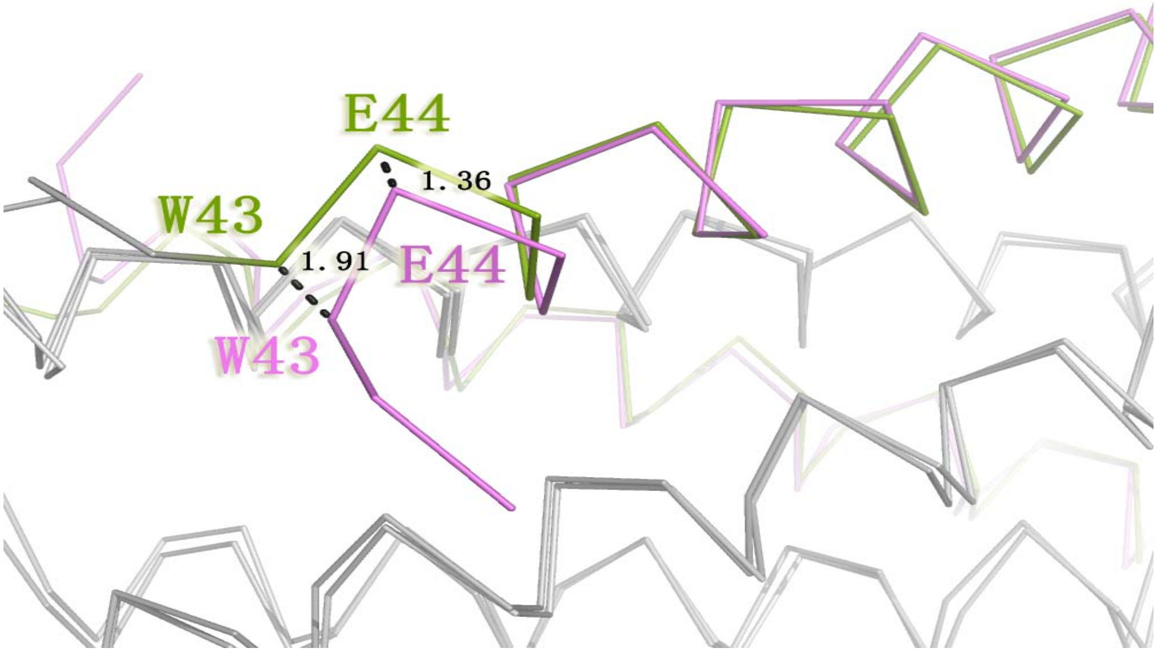

Figure S3| **Different structural characteristics of AP2 and AP3. AP2 and AP3 are shown in ribbon**. Colored identically, AP2 is in split pea, and AP3 is in violet. The interaction residues are shown in stick representation and are properly labeled in separate colors. Salt bridges are shown in dark grey dashed lines, and the unit of bond distance is angstrom.

a

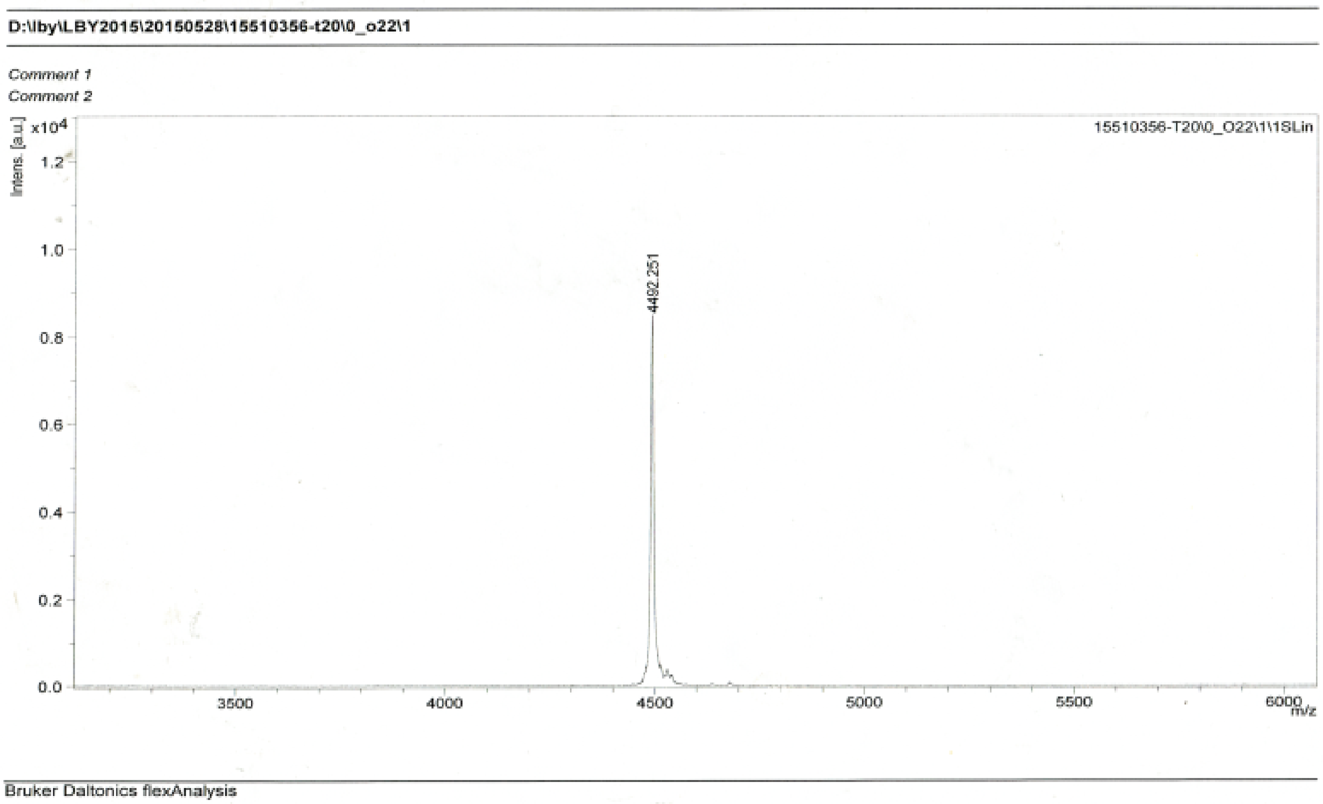

b

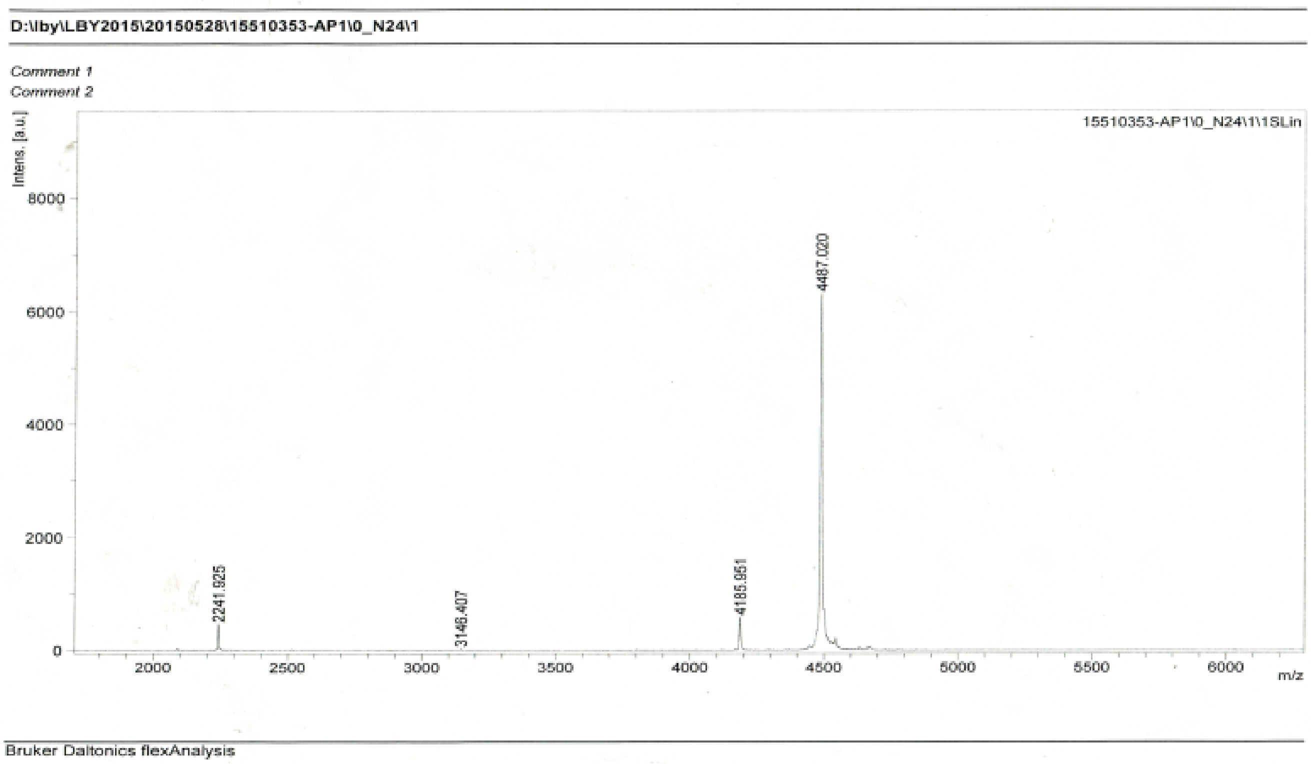

Figure S4 | Masses of T20 (a), AP1 (b) are determined by MALDI-TOF-MS. The X axis represents m/z, Y axis represents intensity.

a

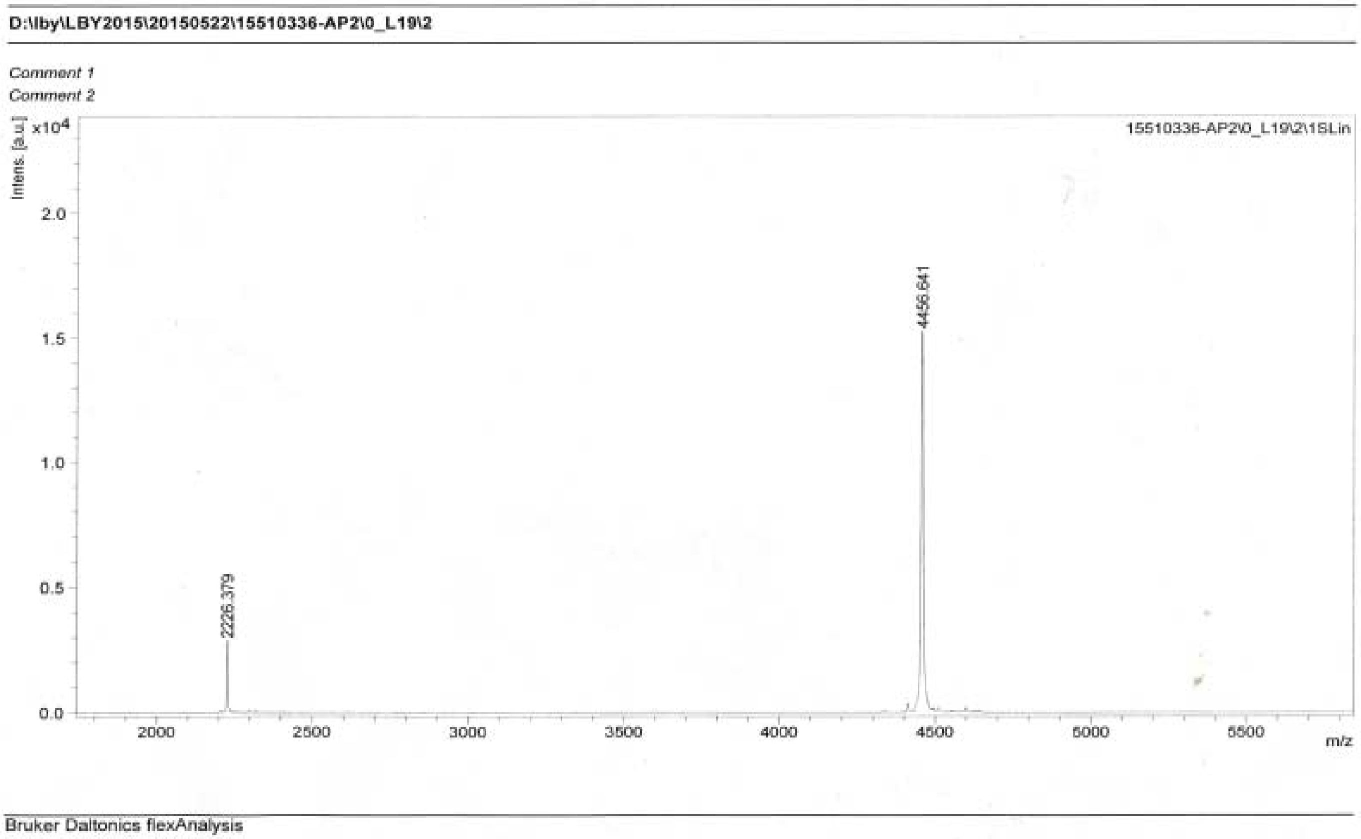

b

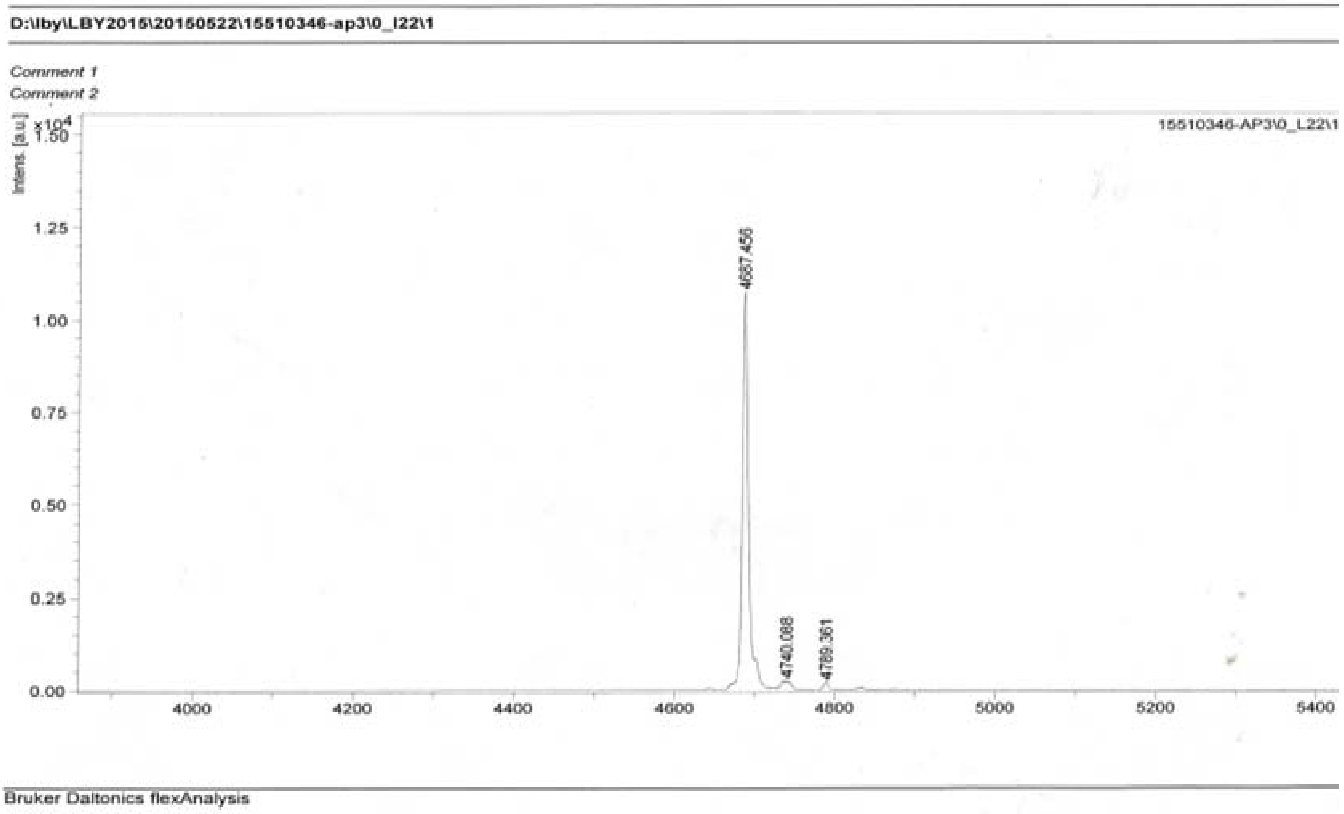

Figure S5 | Masses of AP2 (a), AP3 (b) are determined by MALDI-TOF-MS. The X axis represents m/z, Y axis represents intensity.
